# Structure–Activity Relationships and Transcriptomic Analysis of Hypoxia-Inducible Factor Prolyl Hydroxylase Inhibitors

**DOI:** 10.3390/antiox11020220

**Published:** 2022-01-24

**Authors:** Andrey A. Poloznikov, Sergey V. Nikulin, Dmitry M. Hushpulian, Anna Yu. Khristichenko, Andrey I. Osipyants, Andrey F. Asachenko, Olga V. Shurupova, Svyatoslav S. Savin, Sue H. Lee, Irina N. Gaisina, Gregory R. J. Thatcher, Anthony Narciso, Eric P. Chang, Sergey V. Kazakov, Nancy Krucher, Vladimir I. Tishkov, Bobby Thomas, Irina G. Gazaryan

**Affiliations:** 1 Faculty of Biology and Biotechnologies, Higher School of Economics, 101000 Moscow, Russia; apoloznikov@hse.ru (A.A.P.); snikulin@hse.ru (S.V.N.); 2 School of Biomedicine, Far Eastern Federal University, 690091 Vladivostok, Russia; hushpulian@gmail.com; 3 Dmitry Rogachev National Medical Research Center of Pediatric Hematology, Oncology and Immunology, 117997 Moscow, Russia; hristik14@gmail.com (A.Y.K.); andrey.osipyants@fccho-moscow.ru (A.I.O.); 4 A.V. Topchiev Institute of Petrochemical Synthesis, Russian Academy of Sciences, 119991 Moscow, Russia; aasachenko@ips.ac.ru (A.F.A.); shurupovao@yandex.ru (O.V.S.); 5 Department of Chemical Enzymology, Chemistry Faculty, M. V. Lomonosov Moscow State University, 119192 Moscow, Russia; savinslava@gmail.com (S.S.S.); vitishkov@gmail.com (V.I.T.); 6 Department of Pharmaceutical Sciences and UICentre, College of Pharmacy, University of Illinois, Chicago, IL 60612, USA; suehyunlee12@gmail.com (S.H.L.); igaysina@uic.edu (I.N.G.); 7 Department of Pharmacology & Toxicology, College of Pharmacy, University of Arizona, Tucson, AZ 85721, USA; grjthatcher@arizona.edu; 8 Dyson College of Arts and Sciences, Pace University, New York, NY 10038, USA; anthony.narciso@pace.edu (A.N.); echang@pace.edu (E.P.C.); skazakov@pace.edu (S.V.K.); nkrucher@pace.edu (N.K.); 9 A.N. Bach Institute of Biochemistry, Federal Research Centre “Fundamentals of Biotechnology” of the Russian Academy of Sciences, 119991 Moscow, Russia; 10 Darby Children’s Research Institute, Departments of Pediatrics, Neuroscience and Drug Discovery, Medical University of South Carolina, Charleston, SC 29425, USA; thomasbo@musc.edu

**Keywords:** transcription factor, hypoxia, iron chelation, 2-oxoglutarate dioxygenase, adaptaquin, neuradapt

## Abstract

To evaluate the differences in action of commercially available 2-oxoglutarate mimetics and “branched-tail” oxyquinoline inhibitors of hypoxia-inducible factor prolyl hydroxylase (HIF PHD), the inhibitors’ IC_50_ values in the activation of HIF1 ODD-luciferase reporter were selected for comparative transcriptomics. Structure–activity relationship and computer modeling for the oxyquinoline series of inhibitors led to the identification of novel inhibitors, which were an order of magnitude more active in the reporter assay than roxadustat and vadadustat. Unexpectedly, 2-methyl-substitution in the oxyquinoline core of the best HIF PHD inhibitor was found to be active in the reporter assay and almost equally effective in the pretreatment paradigm of the oxygen-glucose deprivation in vitro model. Comparative transcriptomic analysis of the signaling pathways induced by HIF PHD inhibitors showed high potency of the two novel oxyquinoline inhibitors (#4896-3249 and #5704-0720) at 2 μM concentrations matching the effect of 30 μM roxadustat and 500 μM dimethyl oxalyl glycine in inducing HIF1 and HIF2-linked pathways. The two oxyquinoline inhibitors exerted the same activation of HIF-triggered glycolytic pathways but opposite effects on signaling pathways linked to alternative substrates of HIF PHD 1 and 3, such as p53, NF-κB, and ATF4. This finding can be interpreted as the specificity of the 2-methyl-substitute variant for HIF PHD2.

## 1. Introduction

**Figure 1:**
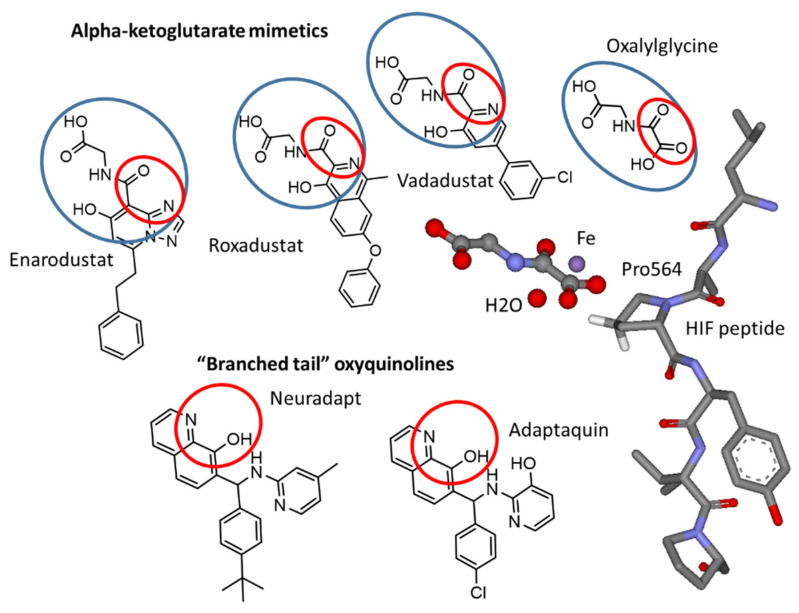
Chemical structures of HIF prolyl hydroxylase inhibitors mimicking αKG (enarodustat, vadadustat, roxadustat, N-oxalyl glycine) and HIF peptide (adaptaquin and neuradapt) binding modes. The oxyquinoline ring provides two iron ligands and the “branched-tails” interfere with HIF peptide binding. Red ovals show iron ligands. Blue ovals show the αKG mimicking part. The arrangement of N-oxalyl glycine (replacing αKG) and HIF peptide with respect to the active site iron in HIF PHD2 (PDB: 3HQR).

The cellular response to hypoxia is executed by the stabilization and activation of hypoxia-inducible factor (HIF), a transcription factor that launches the expression of dozens of genes necessary for survival under low oxygen, with erythropoietin (EPO) being the best-known target. The HIF-triggered program also includes apoptotic/pro-death factors, and depending on the availability of energy resources, the cell may either survive or die. The survival program mainly relies on the cell’s ability to synthesize proteins and enzymes, enhancing anaerobic glycolysis. HIF is a heterodimer, with the dimer stability controlled by an α-subunit (HIFα), which degrades in normoxia. Beta subunits are constitutively-expressed aryl hydrocarbon receptor nuclear translocators. At least three different alpha subunits are known, termed HIF1α, 2α, and 3α. They dimerize with the HIFβ subunit forming the corresponding transcription factors, e.g., HIF1, HIF2, HIF3. HIF1, and HIF2, posessing overlapping and distinct gene targets; HIF2 is considered the key factor regulating EPO production. HIFα stability is regulated by HIF prolyl hydroxylase, α-ketoglutarate iron-dependent dioxygenase (HIF PHD) [1]. This enzyme is at the center of the oxygen-sensing mechanisms in the cell. The active site iron activates oxygen and catalyzes the hydroxylation of Pro402 and/or Pro564 residues in HIFα, making the hydroxylated protein recognizable by the von Hippel–Lindau protein, an adapter in the ubiquitin ligase complex labeling HIFα for subsequent proteasomal degradation. HIFα is continuously produced and degraded under normoxia, and the steady-state level of HIF is very low. In hypoxia, the activity of HIF PHD declines, and HIF levels rise dramatically. HIF PHD is a target [2] for antianemic and antihypoxic drugs under development, shown in Figure 1. There is a large market for these drugs since they could replace recombinant EPO with an oral tablet for the treatment of anemia in dialysis [3] and non-dialysis [4] patients. *N*-hydroxycarbamoyl-based iron chelators such as deferoxamine and ciclopirox could also serve the purpose of HIF PHD inhibition; however, they cannot be considered as medications for chronic anemia because their prolonged use may cause iron deprivation. Therefore, the drugs developed for the treatment of anemia mimic αKG (blue oval in Figure 1) with a freely rotating iron ligand (carbonyl oxygen) and nitrogen atom in a heterocycle (iron ligands shown in red oval in Figure 1). Such combination provides iron coordination but not chelation. The core chemical scaffold is very different and varies from one heterocycle (e.g., pyridine in vadadustat) to fused heterocycle rings (e.g., isoquinoline in roxadustat and triazolopyridine in enarodustat). Only the two latter drugs, enarodustat (Japan Tobacco Inc., Tokyo, Japan) [5] and roxadustat (FG-4592) (FibroGen Inc., San-Francisco, CA, USA), are approved for use in Japan, China, Chile, and South Korea. FDA decision on vadadustat (Akebia Therapeutics Inc., Cambridge, MA, USA) is expected in March 2022. 

All approved drugs and those close to approval have a common pharmacophore motif that includes heterocyclic nitrogen adjacent to the carbon with carboxamide-bound glycine such as the one found in *N*-oxalyl glycine, an exact mimic of the enzyme substrate, α-ketoglutarate (αKG), and as such, an inhibitor of all αKG-dependent Fe-dioxygenases.

The non-specificity of PHD inhibiting drugs can originate from the existence of three HIF prolyl hydroxylases (PHD1, PHD2, PHD3) that regulate HIFα stability. HIF PHD1 and HIF PHD2 are expressed in normoxia, while HIF PHD3 is induced by hypoxia. The developed HIF prolyl hydroxylase pan-inhibitors target all three enzymes because of the high similarity in their active site structure. HIF PHDs have numerous client substrates in addition to HIF itself [6]. Hence, inhibiting HIF PHDs will exert far more profound effects than just by infusing EPO. This could create an additional benefit since EPO-resistant cases also respond to treatment with roxadustat [7] but also may give rise to off-target effects since not all HIF PHD substrates are currently known.

The difference between the two groups of inhibitors shown in Figure 1 is in their ability to chelate iron, which is significant for oxyquinoline-type inhibitors with an estimated dissociation constant of 100–200 nM [8]. In this work, we addressed (a) the plausible role of pyridine nitrogen in the “branched-tail” on drug specificity using structure–activity relationships in the HIF1 ODD-luc reporter assay and computer modeling, and (b) the effect of 2-methyl substitution in the oxyquinoline ring on the inhibitor’s specificity, utilizing comparative transcriptomic analysis.

## 2. Materials and Methods

Roxadustat (FG-4592), vadadustat, and dimethyl oxalylglycine (DMOG) were obtained from Cayman Chemicals (United States). Branched-tail oxyquinolines were purchased from ChemDiv (Moscow, Russia) or synthesized using the established protocol [9]; the minimum purity guaranteed by the supplier was 90%, and all purchased compounds were provided with NMR spectra. 5-Cl-Neuradapt was custom synthesized with >98% purity (see Supplementary Materials Figure S1 for the set of LC-MS and NMR spectra). All other materials and reagents were purchased from commercial vendors as indicated.

The reporter assay on the HIF1 ODD-luc/SH-SY5Y cell line was performed as described in [10]. Drugs were freshly prepared as 10 mM stock solutions in DMSO, and right before the experiment were diluted to 50×-fold to the final concentration in the well, so each well with 100 μL medium was inoculated with 2 μL of the drug solution. Cells were incubated for 3 h and then lysed and assayed for luciferase activity [8]. To evaluate the effect of iron, the cells were loaded with the corresponding concentrations of ferrous sulfate in the medium for 20 min before the drug’s addition. All experiments were performed in triplicate. The fold activation with respect to the background luminescence in the presence 2 μL DMSO aliquot was plotted against drug concentrations. The results are shown as average ± SD.

Computer modeling (docking of drug candidates) was performed as described in [10]. Docking experiments were performed using the CDOCKER algorithm as implemented in the Discovery studio 3.0 software suite (Accelrys, San Diego, CA, USA), followed by force field minimization and binding energy calculations using a crystal structure of HIF PHD2 (PDB: 2G19). Docking method validation was performed by RMSD assessment of extraction and docking of the original ligand, *N*-[(4-hydroxy-8-iodoisoquinolin)-3-yl]glycine. Fixed constraints were applied to protein residues. All ligands were imported and minimized using the ‘Prepare ligands’ protocol that applies a force field, adds hydrogens, and creates compounds’ enantiomers. Force-field minimization was carried out using the molecular mechanics algorithm CHARMm, as implemented in Discovery Studio 3.0. Only poses with correct iron coordination were considered.

For oxygen-glucose deprivation (OGD) studies, 40,000 SH-SY5Y cells were plated in each well of 96-well plates. After overnight incubation, cells were treated with 1 and 3 μM concentrations of drugs for 24 h prior to OGD, at the onset of OGD, and at the onset of reperfusion. The compounds’ toxicity at the 1, 5, and 8 μM concentrations was assessed at 48 h. Oxygen glucose deprivation was performed as follows [11]. The medium was replaced with DMEM containing no glucose, and then the cells were transferred to a sealed hypoxic chamber with 5% CO_2_-95% N_2_ atmosphere for 2 h. After 2 h incubation, the cells were removed from the hypoxic chamber and resupplied with a regular growth media for reperfusion/recovery. After 24 h incubation, cell viability was quantified using an MTT (4,5-dimethylthiazol-2-yl)-2,5-diphenyltetrazolium bromide, Sigma) assay. Data show the mean and SEM normalized to control (*n* = 6): *p* < 0.05 versus control insult (*n* = 6/group), by a one-way ANOVA Dunnett’s test.

Transcriptomic microanalysis was performed as described in [12]. The human neuroblastoma SH-SY5Y cell line was grown on DMEM/F12 (Paneco Ltd., Moscow, Russia) containing 10% FBS (Thermo Fisher Scientific Inc., Waltham, MA, USA), and the cells were plated in 6-well plates (TPP, Russia) at a cell/well density of 500,000 the day before the drug treatment. The drugs—2 μM oxyquinoline(s), 30 μM (roxadustat), and 0.5 mM DMOG—were added to the cells in triplicate, and the cells were incubated for 5 h. The control wells contained 4 μL DMSO. The hypoxic conditions were created by incubation in 1% O_2_, 5% CO_2_, and 94% N_2_ atmosphere for 2 h (the medium was incubated under the same conditions for 16 h before addition to the cells). After 2 h hypoxia, the cells were returned to the original incubation conditions in a CO_2_ incubator and kept for another 5 h. After incubation, the cells were lysed with the Qiazol reagent (Qiagen N.V., Venlo, The Netherlands). The RNA was isolated using the RNeasy Micro Kit (Qiagen); the qualitative and quantitative analysis of the RNA was performed as described earlier [13]. The RNA integrity number (RIN) values for all the samples were above 9.5. All steps of the sample preparation, hybridization, washing, staining, and microchip scanning were performed as described before [14]. The mRNA expression profile was analyzed using the Gene Chip Human Transcriptome Array 2.0 (Thermo Fisher Scientific Inc., Waltham, MA, USA). The CEL files obtained during microchip scanning were treated with the Transcriptome Analysis Console 2.0 application (Thermo Fisher Scientific-Affymetrix, Santa Clara, CA, USA). Unannotated probe sets were excluded from the analysis. The results obtained were statistically processed using the TAC 4.0 software (Thermo Fisher Scientific). The statistical reliability for the differences in the expression levels between the intact cells and cells treated with the studied compounds was evaluated using the monofactorial analysis of variance (ANOVA) with the Benjamini–Hochberg correction. The significance threshold was 0.05.

## 3. Results and Discussion

### 3.1. Comparison of HIF PHD Inhibitors in HIF1 ODD-luc Reporter Assay

**Figure 2:**
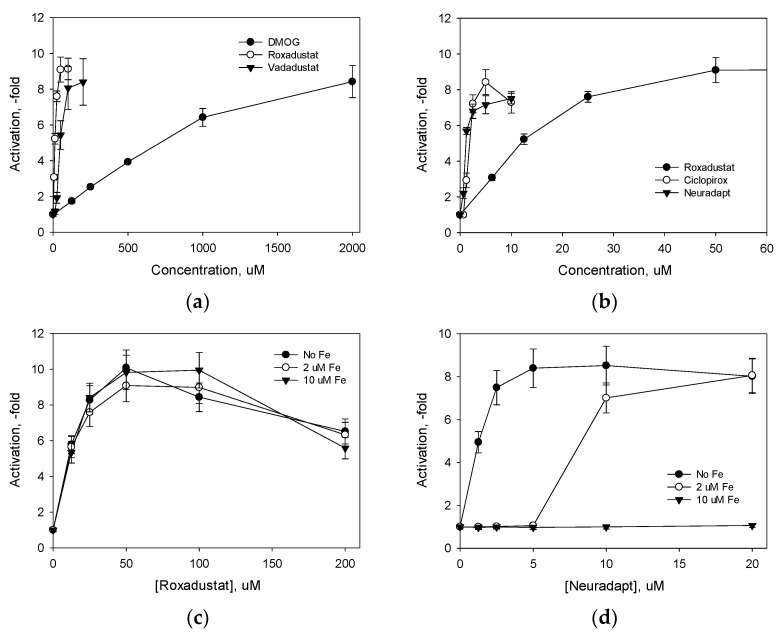
Potency of HIF prolyl hydroxylase inhibitors in HIF1 ODD-luc reporter assay (**a**,**b**), the effect of exogenous iron on reporter activation by roxadustat (**c**), and neuradapt (**d**). Assay conditions under Materials and Methods.

The HIF1 ODD-luc reporter assay employs a cell line stably expressing a reporter vector providing the continuous production of an oxygen-dependent domain of HIF1α fused via its C-terminus to N-terminus of firefly luciferase. The steady-state level of the fusion protein depends on the ratio of the rates of its production (p_cmv_ promoter) and degradation upon hydroxylation, binding to VHL and proteasomal degradation. As we showed before [8], the enzyme step is rate-limiting in the fusion protein degradation, and the steady-state level of HIF1 ODD-luc fusion is determined by the activity of the intracellular pool of all three HIF PHD enzymes. Enzyme inhibition results in an immediate response in the form of luciferase fusion accumulation. The performance of the fusion reporter is equivalent to monitoring the actual fate of intracellular HIFs upon enzyme inhibition. The comparison of HIF PHD inhibitors in the HIF1 ODD-luc fusion reporter assay in the neuroblastoma cell line demonstrated the micromolar potency of commercially available HIF PHD inhibitors in comparison to the millimolar potency of dimethyl oxalyl glycine (DMOG), a mimic of the enzyme substrate αKG (Figure 2a). However, both vadadustat and roxadustat worked in a very high micromolar range (IC_50_ above 30 μM). The drugs mimicking αKG are competitive HIF PHD inhibitors and hence, compete with millimolar intracellular αKG for the target enzyme. Such competition explains the need for high micromolar concentrations of the drugs (roxadustat, vadadustat, etc.) despite the fact that these drugs exhibit excellent nanomolar inhibition constants in the in vitro enzymatic assay [15]. The use of high concentrations of the αKG mimetic drugs to overcome the competition from the intracellular αKG may result in targeting other αKG-dependent pathways that are unrelated to αKG-dependent dioxygenases, such as kynurenine-oxoglutarate transaminase and αKG dehydrogenase E1. In fact, DMOG is known to inhibit the αKG dehydrogenase complex and oxygen consumption by mitochondria [16]. This plausible break in specificity could be a reason for concerns on the safety of HIF PHD inhibitor drugs in comparison with the use of recombinant EPO [17].

On the other hand, HIF PHD inhibitors mimicking αKG binding are not expected to chelate iron in solution because the pharmacophore part responsible for the active site iron coordination contains two freely rotating iron ligands (shown in a red circle inside the blue circle in Figure 1).

Besides these structural considerations, vadadustat, roxadustat, and other drugs of this class were optimized in the homogeneous enzyme assay in the presence of 50 μM ferrous salts. This selection resulted in drugs with a high affinity for the active site residues rather than the active site iron. This is in contrast to oxyquinoline and other aromatic inhibitors, having adjacent iron ligands on the same aromatic core (shown in a red circle in Figure 1), making a strong chelation motif and resulting in potent HIF PHD inhibition (Figure 2b). However, the “branched-tail” oxyquinolines under study are worse iron chelators than ciclopirox (*K_D_* of 100–200 nM versus 50 nM for ciclopirox) but are two–three-fold more potent activators than ciclopirox and more than an order of magnitude better activators than roxadustat in HIF ODD-luc reporter assays (Figure 2b). The structure–activity relationships described before for this group of oxyquinolines [8] point to the specific character of their interaction with HIF PHD. Nevertheless, the presence of free iron will diminish the number of chelator drugs available for HIF PHD inhibition. The reporter assay in the presence of free iron (II) sulfate (Figure 2c,d) shows the interference of free iron with the reporter activation, which is not observed for roxadustat. Importantly, the concentration of free iron was equal to or exceeded the IC_50_ (1 μM) for the best hit (compound 4896-3212, named neuradapt), but was three times lower than the IC_50_ for roxadustat (30 μM). Based on the results obtained in the HIF1 ODD-luc reporter assay, one may conclude that iron chelating inhibitors are not suitable for anemic patients, but such inhibitors may present an additional benefit for conditions of oxidative stress associated with the release of iron, such as ferroptosis [18].

### 3.2. Refining Structure-Activity Relationships for “Branched-Tail” Oxyquinoline Inhibitors

**Figure 3:**
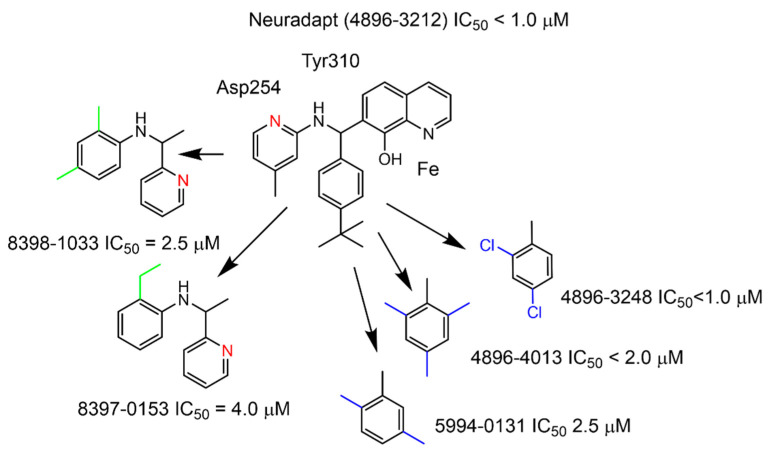
Structure–activity relationships derived from the titration experiments. All compounds are commercially available from ChemDiv (Skolkovo, Russia).

The “branched-tail” of adaptaquin and neuradapt-type inhibitors contains a pyridine moiety in the “tail”, which could also provide an iron coordination ligand similar to the previously described metal chelators [19]. However, this type of coordination is impossible for the seven substituted oxyquinolines used in this study, since the length of the linker is too short to reach the iron atom coordinated by the nitrogen and hydroxyl groups in the oxyquinoline core. We tested the activity of several variants, switching the pyridine to a phenyl ring between the “tail branches” (Supplementary Figure S2) and found that the IC_50_ of reporter activation was sensitive to the structural change (Figure 3).

**Figure 4:**
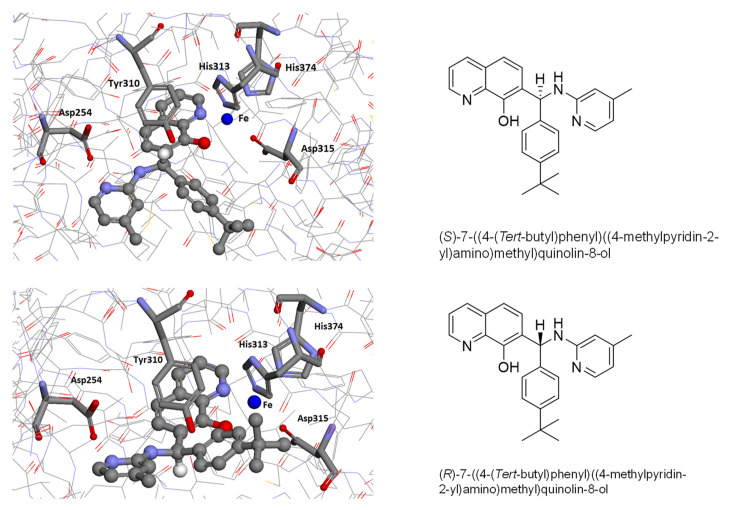
Docking of neuradapt enantiomers into HIF PHD2 crystal structure (PDB: 2G19). Asp254 interacts with the pyridine nitrogen, and Tyr 310 restricts rotation of pyridine and phenyl rings in the “branched-tail”(see Table S1 in Supplement for docking energy values).

Docking of both enantiomers of neuradapt (compound 4896-3212) positioned the nitrogen atom of pyridine in proximity with Asp254 residue in the enzyme active center, suggesting the possibility of hydrogen bonding between the two (Figure 4).

The structure–activity relationship depicted in Figure 3 and derived from the titration experiments for the studied compounds (Figure S2 in Supplementary Materials) can be explained based on the docking results of neuradapt enantiomers shown in Figure 4, since hexacoordinated iron imposes the major restriction on the position of the oxyquinoline ring and permits the rotation of the branched-tail only. Despite the sufficient room for *para*-isobutyl substitution in the phenyl ring in neuradapt (compound 4896-3212), the rotation of the phenyl ring has spatial restrictions when both *ortho*-positions (Compound 4896-4013), or *ortho*- and *meta*-substituents (Compound 5994-0131), are present. Based on the docking of the neuradapt enantiomers, one may expect interference from *ortho*- and especially *meta*-substituents in the phenyl ring for the (*R*)-enantiomer form of the inhibitors. Whereas, when the pyridine is replaced with phenyl, the interaction with Asp254 is lost, and in addition, the presence of ethyl group in the *ortho*-position (Compound 8397-0153) interferes with free rotation, causing a drop in affinity for the enzyme.

**Figure 5:**
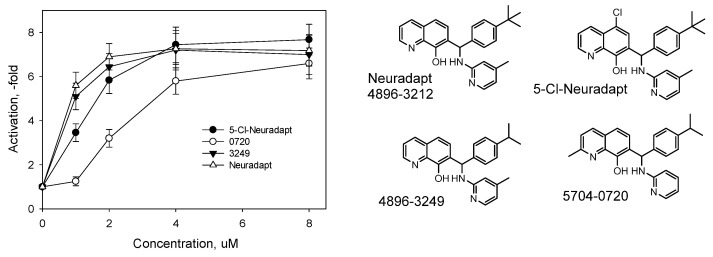
Effect of substitutions in the second and fifth position of the oxyquinoline core on HIF1 ODD-luc reporter activation. 5-Cl-Neuradapt was custom synthesized (See Figure S1). Other compounds are commercially available from ChemDiv (Skolkovo, Russia).

To study the possibility of additional substitutions in the oxyquinoline ring, we explored the addition of chlorine in the fifth position and methyl group in the second position. As seen from the data in Figure 5, 5-chloro-neuradapt (custom synthesized, see LC, NMR, and MS data in Figure S1) is almost as active as the original compound.

In our early work [8], all 2-methyl-substituted oxyquinolines were inactive at low micromolar concentrations and activated the reporter only at the concentration above 5 μM, the range that is typical for non-specific iron chelators. Since neuradapt (compound 4896-3212) was structurally optimized to exhibit submicromolar IC_50_ values, we decided to explore the effect of 2-methyl-substitution in the oxyquinoline on the activation parameters in HIF1 ODD-luc assay. However, we decided to “ease” the neuradapt structure with respect to rotation restrictions in the “branched-tail” and to (a) replace the isobutyl-group with isopropyl in the *para*-position of the phenyl ring (compound 4896-3249) and (b) remove 4-methyl substitution from the pyridine branch (compound 5704-0720). The isopropyl variant of neuradapt (compound 4896-3249) was equally active (Figure 5), and the introduced 2-methyl substitution slightly increased the IC_50_ value (from 1 to 2 μM). The docking of a 2-methyl variant into a PHD2 crystal structure gives similar values of docking energy as for compound 4896-3249 (Table S1), despite the tight fit of the 2-methyl-group on the oxyquinoline ring with respect to the active site pocket Leu343 (Figure S3). Overall, small substitutions in the second and fifth positions of the optimized structure of a “branched-tail” oxyquinoline inhibitor did not significantly compromise their reporter activation capacity.

### 3.3. “Branched-Tail” Oxyquinoline Inhibitors in Oxygen Glucose Deprivation (OGD) Model

**Figure 6:**
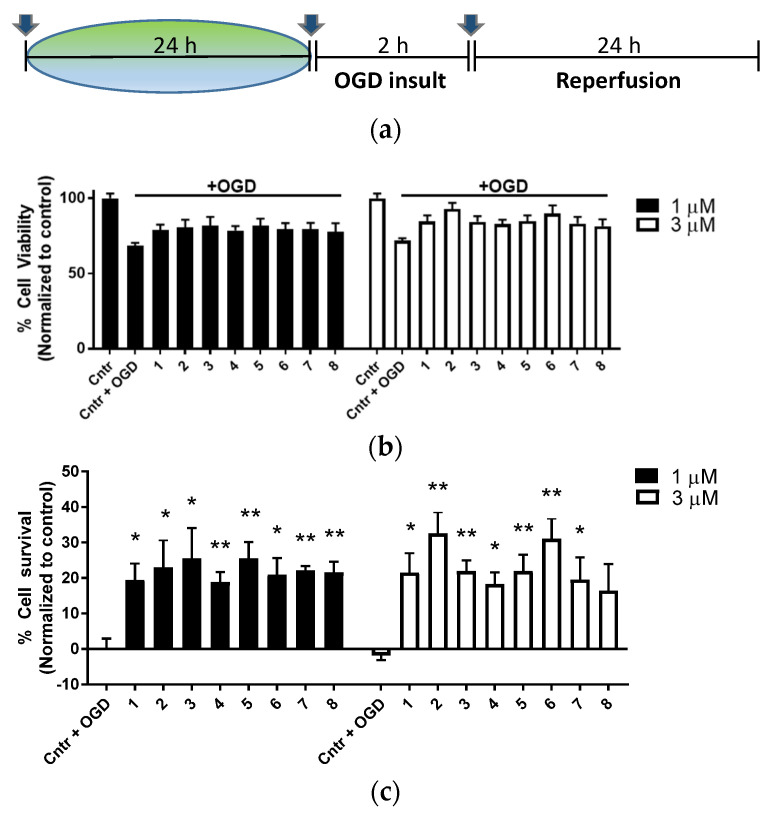

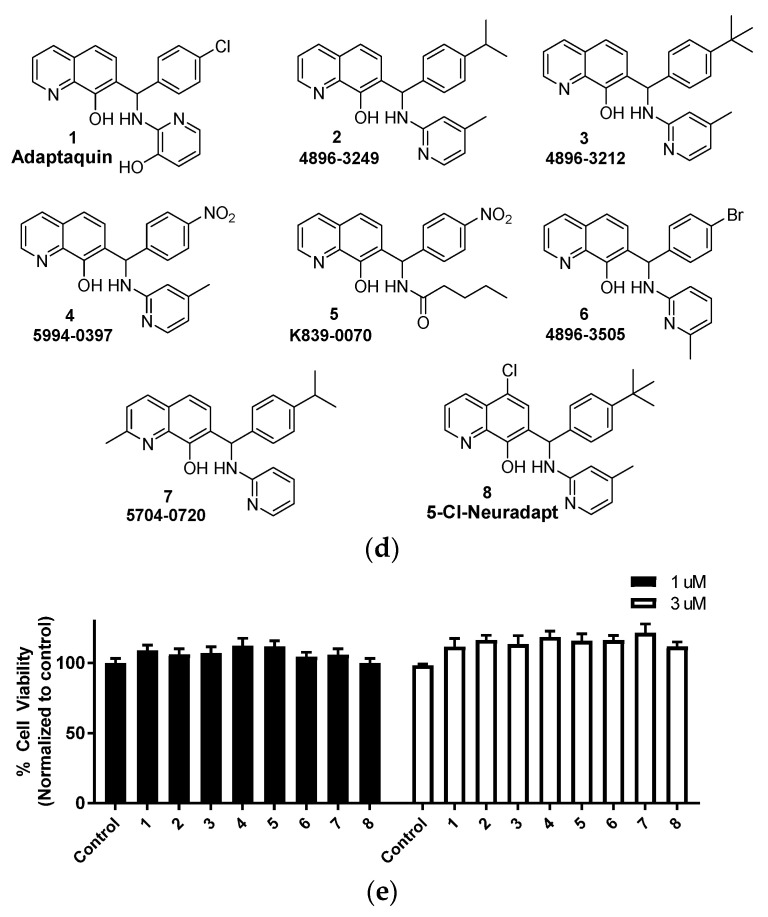
HIF PHD inhibitors protect SH-SY5Y cells subject to OGD: timeline (**a**); cell viability measured after 24 h of incubation using the MTT assay (**b**); 24 h pretreatment (**c**). Data show mean and SEM normalized to control (*n* = 6): * *p* < 0.05, ** *p* < 0.01 versus insult by a one-way ANOVA test; HIF PHD inhibitors structures (**d**); The absence of toxicity of the studied compounds at 48 h incubation (**e**).

The oxygen deprivation model (OGD) was performed using the human neuroblastoma SH-SY5Y cell line. We chose adaptaquin as a control compound for the group of branched oxyquinolines since it showed neuroprotection in the post-treatment of hemorrhagic stroke in vivo model [20] and oxidative stress in vitro model at 1 μM concentration [21,22]. The experimental design was the same as we used before for the drugs targeting HIF PHD, Nrf2, and HDAC6, which showed almost complete neuroprotection at 24 h pretreatment in the range of concentrations matching the IC_50_ in the HIF1 ODD-luc and Neh2-luc reporter assays [11]. The branched oxyquinoline drugs were added either 24 h before the onset of OGD, at the onset of ischemia induced by OGD insult, or with the reperfusion media at the end of OGD, as described in Methods and shown in Figure 6a.

Only pretreatment showed partial neuroprotection (Figure 6b,c), with the highest protection for compound 2 (#4896-3249) at 3 μM by increasing viability from 70 to 90% (Figure 6c), whereas post-treatment had no effect (Figure S4a,b). In the pretreatment regimen, all studied compounds at 1–3 μM provided 20–30% neuroprotection, similarly to adaptaquin, within the experimental error. The studied oxyquinoline inhibitors have no additional activities compared to the compounds with tri-partite activities from [11], and thus, one may conclude that neuroprotection in the OGD model benefits from additional activation of the Nrf2-driven program.

Only a number of reports in the literature on the application of HIF prolyl hydroxylase inhibitors deal with OGD modeling: DMOG [23], FG-4497 [24], and IOX3 [25] in vitro and in vivo; and a set of five inhibitors including Bayer85-3934 and four αKG mimetics: DMOG, FG-4592(roxadustat), FG-2216, and GSK1278863, in vitro only [26]. The results obtained in the above-cited reports are not consistent since the in vivo model is not equivalent to the in vitro neuronal or cancer cell line models. DMOG is not specific for HIF PHD and targets all αKG dependent enzymes. The post-treatment protection observed in [23] can be explained by the DMOG-induced inhibition of mitochondrial respiration as described in [16]. FG-4497, the only inhibitor showing adequate protection in a post-treatment regime, has sulfur replacing oxygen in the tail of roxadustat molecule, and as such, can easily generate oxidation metabolites with additional activities. Pretreatment with IOX3 was neuroprotective in transient focal cerebral ischemia in mice [25]. Ciclopirox, the iron chelator inhibiting HIF PHD and activating HIF1 ODD-luc reporter (Figure 2b), is unlikely to branch oxyquinolines in this study and the five inhibitors studied in [26], and is also neuroprotective in the post-treatment regimen, but due to its demonstrated effect on phosphokinases and cell-cycle related phosphoproteome [27].

The neuroprotective concentrations of the studied oxyquinoline inhibitors in the in vitro OGD model closely match the IC_50_ values determined in the reporter assay. In particular, the oxyquinolines were neuroprotective at 1–3 μM concentration, within the range of their IC_50_ values. The results obtained for oxyquinolines, i.e., the negligible effect of structural variations, and neuroprotection slightly above IC_50_ in the reporter assay, are similar to those for five commercially available HIF PHD inhibitors of various structures: they were protective in PC12 cells and primary rat neurons in concentrations of 30–100 μM [26]. Roxadustat (FG-4592) exhibits an IC_50_ of ca. 25 μM (Figure 2b), and thus, is expected to be protective at concentrations above the IC_50_ value as it was, in fact, observed in [26]. The need for pretreatment with HIF PHD inhibitors could be explained since the execution of the HIF-induced survival program requires glucose supply. In OGD, both oxygen and glucose supply are compromised, making the production of protective proteins/enzymes impossible. In hypoxia, without glucose deprivation, neuradapt was shown to be an effective post-treatment [10].

The study of compounds’ toxicity at prolonged incubation at 8 μM (Figure S2) showed that not all compounds were equally toxic: the least toxic was the newly explored version of neuradapt with substitutions in the second and fifth position (compounds **7** and **8** in Figure 6b). One may speculate that these substitutions made the compounds more specific for the target and thus, minimized the toxicity originating from non-specific side effects. Based on these results, the 2-methyl substituted variant (compound 5704-0720) was chosen for transcriptomic studies in parallel with the closest structural analog, the isopropyl variant of neuradapt (compound 4896-3249), to evaluate the possible differences in their specificity for the target and their impact on downstream signaling pathways.

### 3.4. Comparative Transcriptomic Analysis of HIF Prolyl Hydroxylase Inhibitors

**Figure 7:**
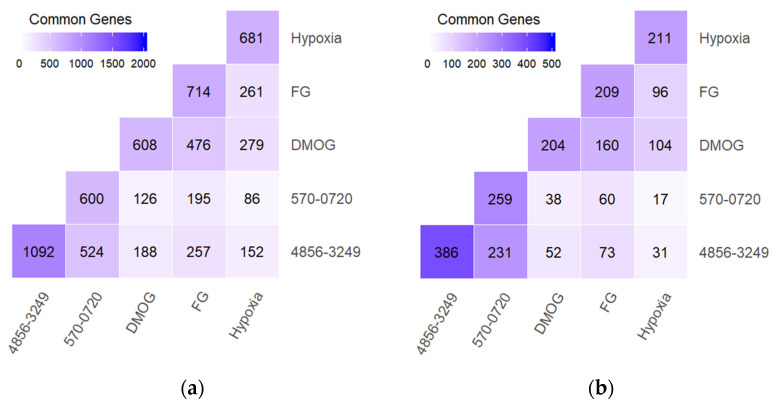
Overlapping genes upon 5 h treatment of human neuroblastoma SH-SY5Y cell line with 0.5 mM DMOG, 30 μM roxadustat (shown as FG), 2 μM compounds #4896-3249 and #5704-0720, and 2 h hypoxic treatment followed by 5 h incubation. (**a**) Activation, (**b**) Repression. 1.5-fold change cut-off. Experimental details under Materials and Methods.

The choice of HIF PHD inhibitors’ concentrations for transcriptomic analysis was based on the IC_50_ values for HIF1 ODD-luc reporter activation (30 μM roxadustat and 0.5 mM DMOG); however, both oxyquinoline inhibitors were used at 2 μM, even though the 2-methyl variant of neuradapt was ca. two-fold less potent in the reporter assay (Figure 5). The incubation was performed for 5 h to focus on immediate early genes, to presumably see the effect of enzyme inhibition on direct substrates and their signaling pathways. As shown in Figure 7, five-hour incubation at concentrations equal to the IC_50_ for roxadustat, DMOG and the 2-methyl variant of neuradapt (compound 0720) upregulate approximately 600–700 genes (Figure 7a) and downregulate 200–260 genes at the 1.5-fold cut-off (Figure 7b). 

More than 30% of the genes upregulated by roxadustat (FG) and DMOG overlap with those induced by hypoxia. For downregulated genes, the overlap between hypoxia and roxadustat or DMOG is higher, nearly 50%. The highest overlap is observed for the couple roxadustat and DMOG: 40% of the upregulated genes and 80% of downregulated genes overlap. This similarity in gene signatures is the result of the structural similarity of the two drugs. Roxadustat design has a similar binding mode to DMOG, which is an exact mimic of αKG. The overlap between the two inhibitors is higher than the overlap of either one with hypoxia, meaning that both have additional targets not related to oxygen-dependent enzymes.

Branched-tail oxyquinolines show much lower overlapping scores with the other drugs and hypoxic treatment, but amongst the three, the overlapping score with roxadustat is the highest. Branched-tail oxyquinolines have a rather bulky structure that prevents them from targeting other known enzymes of the αKG iron dioxygenase family [8], and this may explain the lower overlapping score. Overall, the inhibitor activity in the reporter assay seems to be a reliable way to predict the drug potency in activating gene expression.

**Figure 8:**
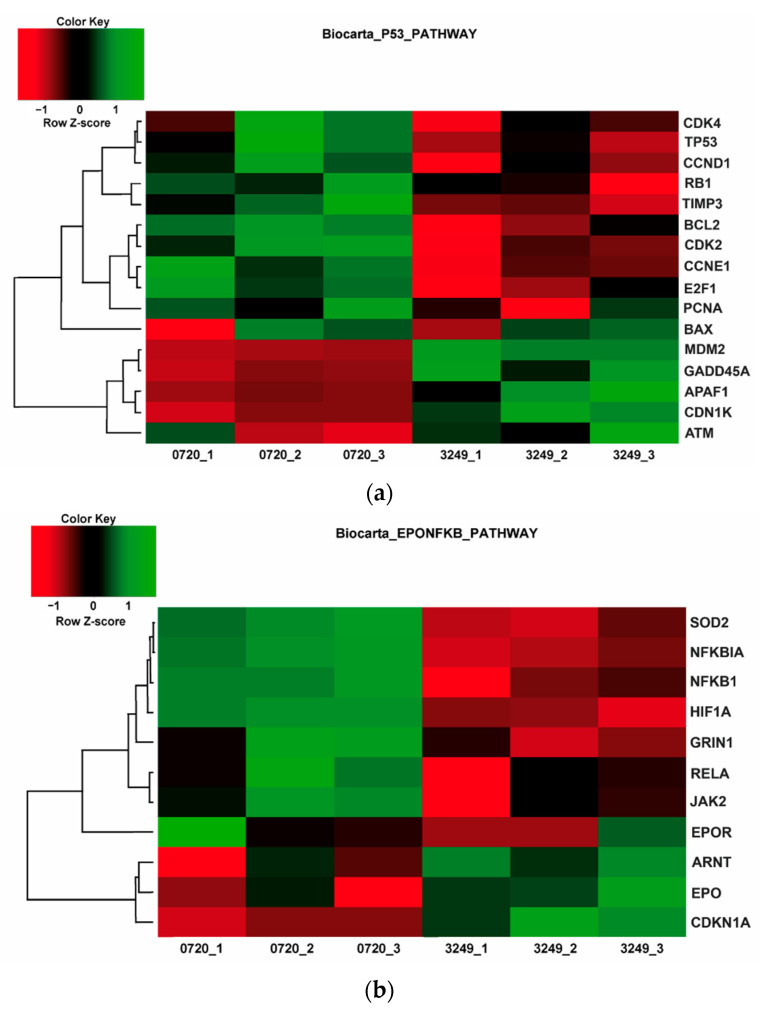
Opposite pattern on heatmaps for Biocarta p53 (**a**) and EPO/NF-κB (**b**) pathways in response to the treatment with compounds #4896-3249 and #5704-0720. All triplicates for each compound are shown. Experimental details under Materials and Methods.

The effect of 2-methyl substitution is clearly visible in the reduced number of overlapped genes with roxadustat (ca. by 20%) in comparison to isopropyl-neuradapt (compound 4896-3249). However, the overlapping pattern for the 2-methyl-substituted variant parallels the one for isopropyl-neuradapt. Nevertheless, there is a difference between the two oxyquinoline inhibitors in gene expression for Hsp70, integrin beta 8, metalothioneins, hyaluronate synthase, and heme oxygenase 1. Comparison of the heat maps shows the similarity between the two compounds in glycolysis and the HIF1 pathway (see Figure S5 in Supplementary Materials), but clear differences in the 28 (BIOCARTA), 3 (KEGG), 5 (PID), and 33 (REACTOME) metabolic and signaling pathways. As shown in Figure 8, the most pronounced is the difference between the p53 and EPO/NF-κB pathways. HIF PHD3 is known to hydroxylate proline in p53 [28] as well as EPO receptors, whereas HIF PHD1 controls the activation of NF-κB [29]. Hence, the observed lower number of overlapping genes for the 2-methyl substituted variant could be the result of its preference for the HIF PHD2 enzyme over the other two enzyme forms.

The comparison of heatmaps of all drugs used in this study and 2 h hypoxia show a strong effect of isopropyl-neuradapt (compound 4896-3249), which inhibits several pro-death pathways with ATF4 being one of them (see Figure S6). Transcription factor ATF4 was shown to be a substrate for HIF PHD3 [30]. Notably, the suppression of ATF4 signaling pathway is weaker for DMOG and roxadustat than for isopropyl-neuradapt (compound 4896-3249), and no suppression of the pathway is observed for the 2-methyl-substituted neurdapat variant (compound 5704-0720). This again points to the interference of the 2-methyl-substitution in the oxyquinoline core with its recognition by the enzyme responsible for the interaction or modification of ATF4 and therefore implicated in the ATF4 signaling pathway.

## 4. Conclusions

It is well-known that HIF prolyl hydroxylase inhibitors are effective in OGD models in a pretreatment paradigm, which is likely linked to the need for the activation of the HIF-triggered antihypoxic defense program. PHD inhibition does not improve locomotor recovery after SCI [31]; however, post-treatment is effective in oxidative stress scenarios. Neuradapt was shown to be neuroprotective in a hypoxia model in the hippocampal neuronal network [10]. Adaptaquin, a branched-tail oxyquinoline inhibitor discovered earlier [8], is neuroprotective in in vivo models of hemorrhagic stroke [20] and Parkinson’s disease [32], and against oxidative stress in vitro models [21,22]. There are studies demonstrating that neuroprotection exerted by adaptaquin in vivo is not HIF dependent [33]. Protection from oxidative death in vitro or from intracerebral hemorrhage in vivo by adaptaquin was associated with suppression of the activity of the pro-death factor ATF4 rather than the activation of an HIF-dependent prosurvival pathway [20]. The comparative transcriptomic studies on various HIF PHD inhibitors point to more specific properties of neuradapt variants towards the target enzyme. Isopropyl-neuradapt (compound 4896-3249) exhibits the enhanced suppression of an ATF4-driven program in comparison to DMOG and roxadustat, whereas its 2-methyl-substituted variant (compound 5704-0720) is inactive (matches to the control levels of the untreated cells Figure S4). The distinct properties of the 2-methyl-substituted variant of neuradapt with respect to p53, NF-κB, and ATF4 signaling programs allows us to speculate that this variant of neuradapt is more specific for HIF PHD2 in comparison to all other studied pan-inhibitors and may be used as a control for pan-inhibitors of the branched-tail oxyquinoline group targeting all three enzyme forms.

With respect to the applicability of the drugs for anemia, from these results, it is obvious that roxadustat is much better suited than drugs such as neuradapt, which exhibit iron chelation activity. The inhibitory properties of the studied oxyquinolines with respect to HIF PHD could be quenched by excess iron, and therefore these drugs cannot be administered together with iron, as typically needed for anemia patients. However, with respect to applications in neurodegenerative diseases where excess iron is harmful, such as in hemorrhagic stroke or Parkinson’s disease, neuradapt will be beneficial due to this property.

## Data Availability

The data sets presented in this study can be found in online repositories. The names of the repository/repositories and accession number(s) can be found below: https://www.ncbi.nlm.nih.gov/geo/query/acc.cgi?acc=GSE189905 (accessed on 19 January 2022).

## Supplementary Materials

The following are available online at https://www.mdpi.com/article/10.3390/antiox11020220/s1, Figure S1: Purity of newly synthesized 5-Cl-Neuradapt: (a) LC-MS chromatograms; (b) NMR spectra; Figure S2: Reporter activation by neuradapt (4896-3212) and its structural variants (see structures in Figure 3). Assay conditions under Materials and Methods; Figure S3: Docking of two enantiomers (A,B) of neuradapt (4898-3212) and two enantiomers (C,D) of 2 methyl variants (5704-0720) into HIF PHD2 binding site (PDB: 2G19). Two histidine residues (313 and 374) and Asp315 residue coordinate iron ion, Leu343 residue controls sterically a radical of the 2-methyl substituent. Figure S4: No protection at post-treatment: (a) treatment at the onset of OGD; (b) treatment at the onset of reperfusion; (c) cell viability at 8 µM of compounds and 48 h incubation without OGD (toxicity); Figure S5: Heatmaps for compounds 4896-3249 and 5704-0720 against control untreated cells show similar pattern and strength of activation of HIF-triggered programs (glycolysis, HIF1 and HIF2); Figure S6: Heatmap for ATF4 signaling pathway comparing all HIF PHD inhibitors tested: (1) control untreated cells; (2) 2 h hypoxia; (3) DMOG; (4) roxadustat; (5) 2-methyl variant (compound 5704-0720); and (6) compound 4896-3249; Table S1: Docking energy for enantiomers of Neuradapt and its analogs; Supplementary Methods: Synthesis Scheme and Protocol.
