# Supplementary material for: Structure–Activity Relationships and Transcriptomic Analysis of Hypoxia-Inducible Factor Prolyl Hydroxylase Inhibitors
Source: Antioxidants (Basel). 2022 Jan 24;11(2):220. doi: 10.3390/antiox11020220 (PMC8868400; doi:10.3390/antiox11020220)

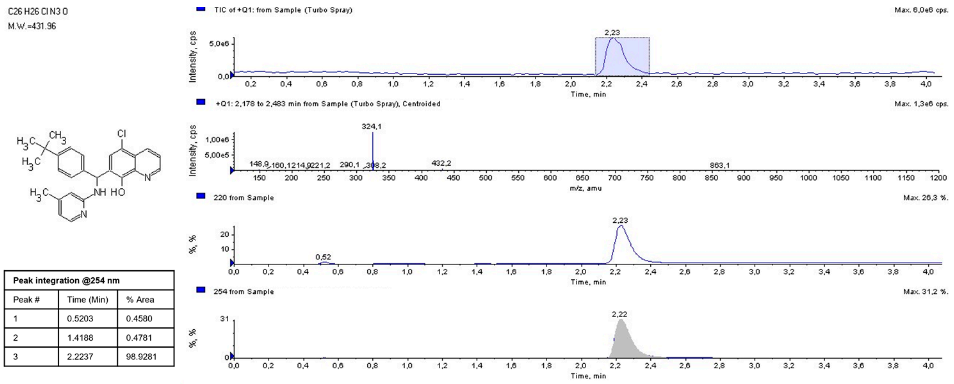

Peak #	Time (Min)	% Area
1	0.5203	0.4580
2	1.4188	0.4781
3	2.2237	98.9281

(**a**)

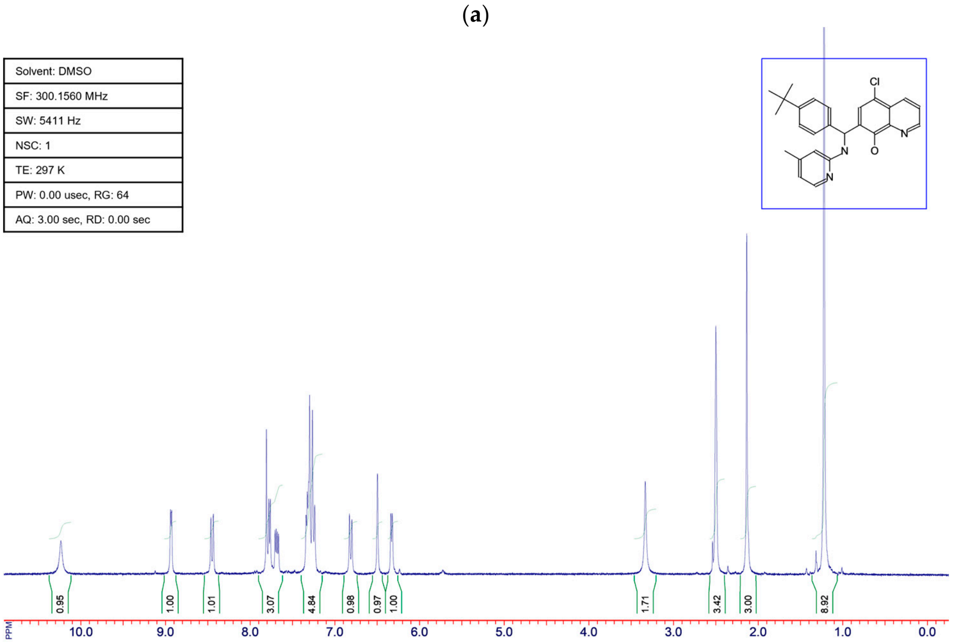

(**b**)

Figure S1. Purity of newly synthesized 5-Cl-Neuradapt: (a) LC-MS chromatograms; (b) NMR spectra.

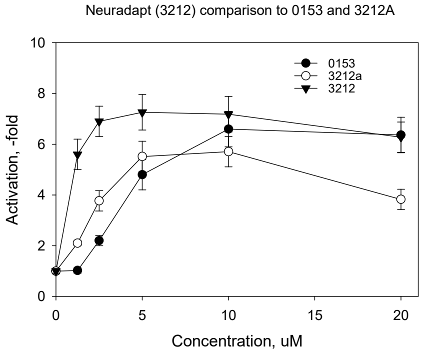

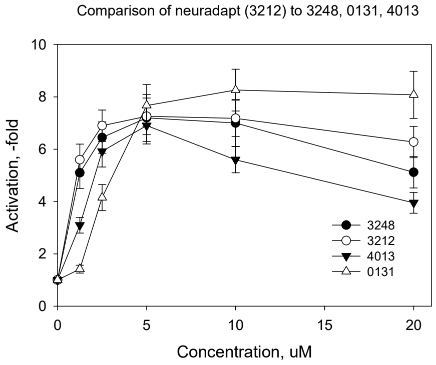

(**a**) (**b**)

Figure S2. Reporter activation by neuradapt (4896-3212) and its structural variants (see structures in Fig.3). Assay conditions under Materials and Methods.

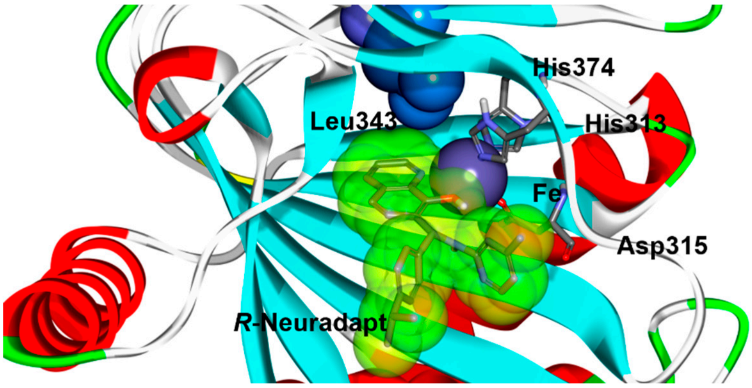

(**a**)

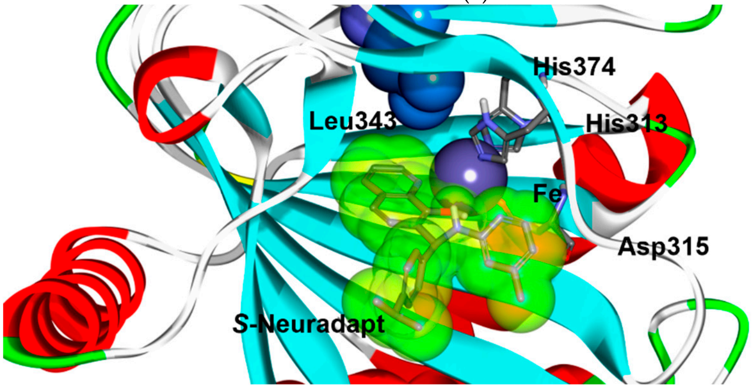

(**b**)

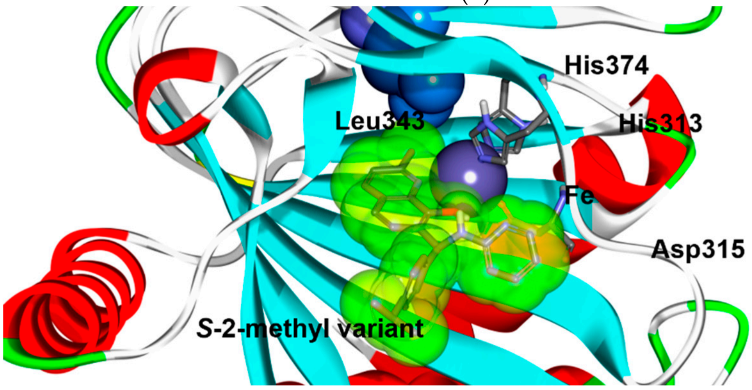

(**c**)

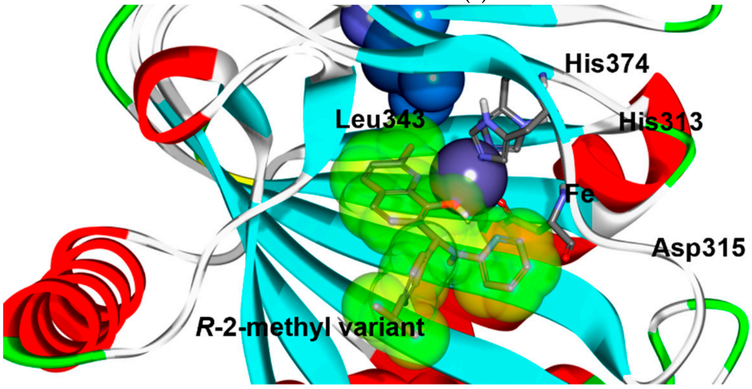

(**d**)

Figure S3. Docking of two enantiomers (A, B) of neuradapt (4898-3212) and two enantiomers (C, D) of 2 methyl variant (5704-0720) into HIF PHD2 binding site (PDB: 2G19). Two histidine residues (313 and 374) and Asp315 residue coordinate iron ion, Leu343 residue controls sterically a radical of 2-methyl substituent.

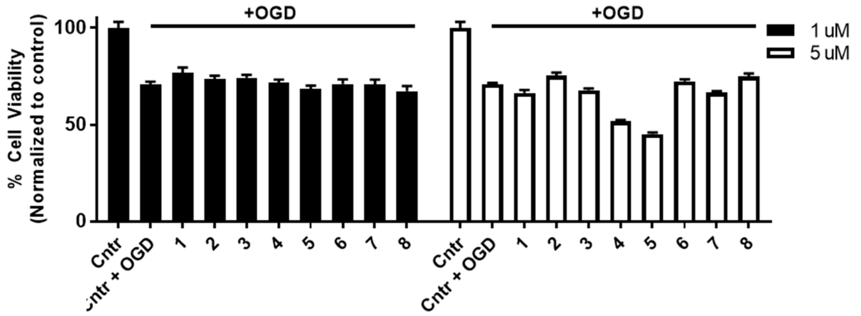

(**a**)

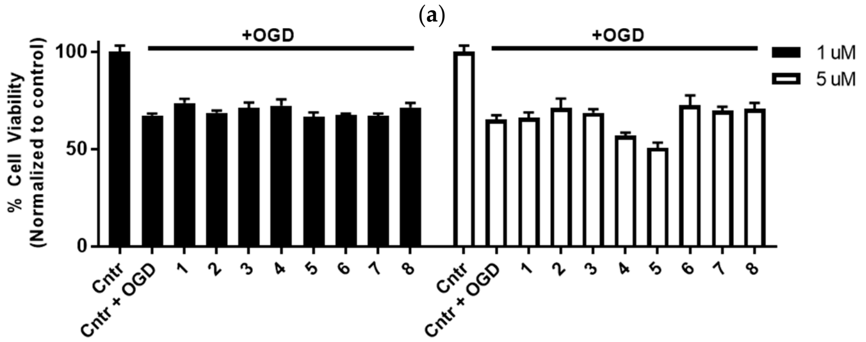

(**b**)

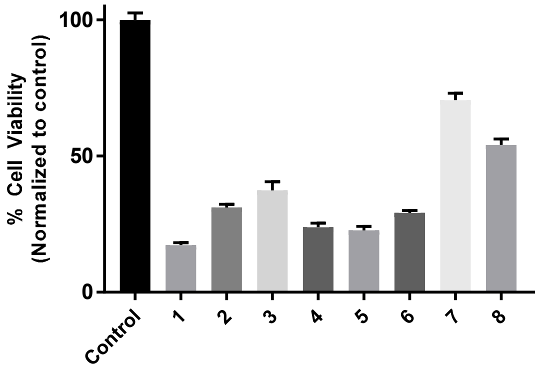

(c)

Figure S4. No protection at post-treatment: (a) treatment at the onset of OGD; (b) treatment at the onset of reperfusion; (c) cell viability at 8 μM of compounds and 48 h incubation without OGD (toxicity).

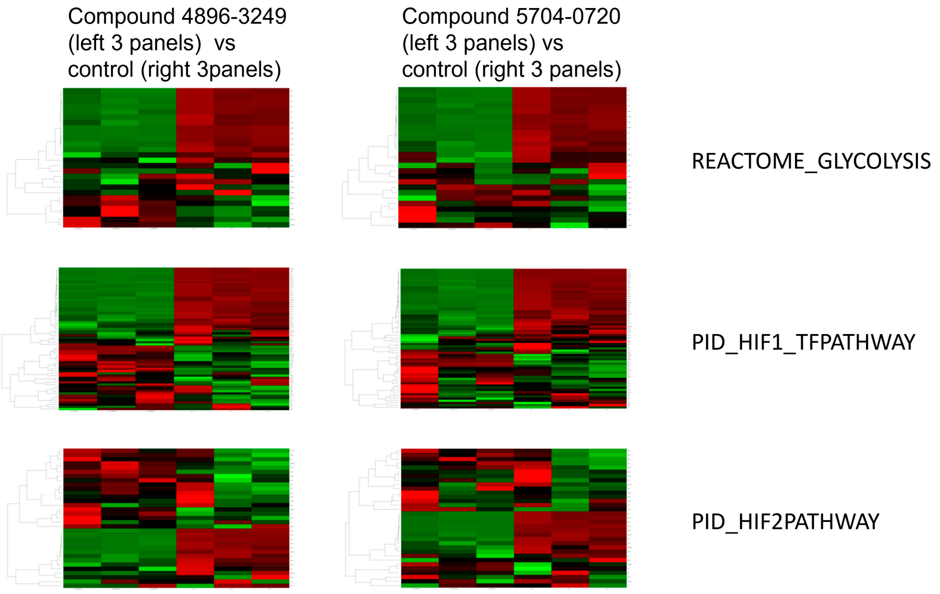

Figure S5. Heatmaps for compounds 4896-3249 and 5704-0720 against control untreated cells show similar pattern and strength of activation of HIF-triggered programs (glycolysis, HIF1 and HIF2).

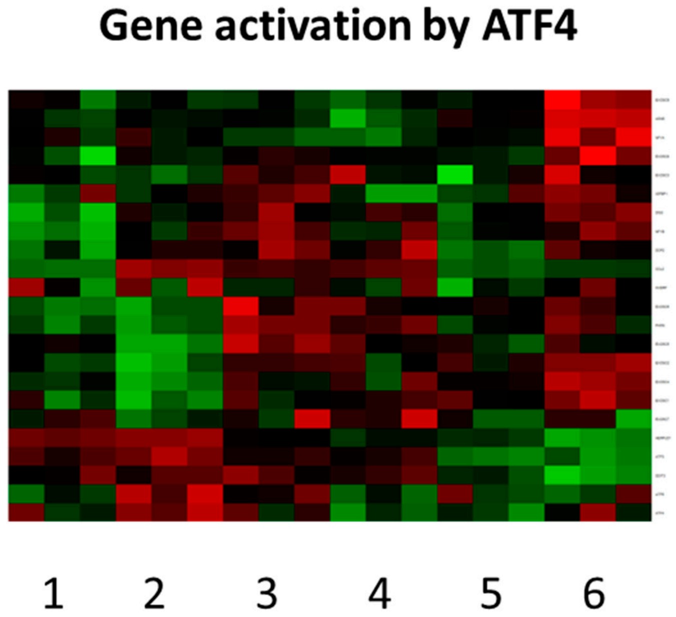

Figure S6. Heatmap for ATF4 signaling pathway comparing all HIF PHD inhibitors tested: (1) control untreated cells; (2) 2 h hypoxia; (3) DMOG; (4) roxadustat; (5) 2-methyl variant (compound 5704-0720); and (6) compound 4896-3249.

Table S1. Docking energy for enantiomers of Neuradapt and its analogs

##	Name	-Cdocker Energy	-Cdocker Interaction Energy
1	Docking validation N-[(4-HYDROXY-8-IODOISOQUINOLIN-3-YL)CARBONYL]GLYCINE	50.1786	57.3668
2	(S)-Neuradapt, enantiomer #1	24.6286	50.339
3	(R)-Neuradapt, enantiomer #2	27.2014	51.4757
4	7-{[(4-METHYLPYRIDIN-2-YL)AMINO][4-(PROPAN-2-YL)PHENYL]METHYL}QUINOLIN-8-OL #1	27.9668	42.9324
5	7-{[(4-METHYLPYRIDIN-2-YL)AMINO][4-(PROPAN-2-YL)PHENYL]METHYL}QUINOLIN-8-OL #2	25.1192	45.8014
6	2-METHYL-7-{[4-(PROPAN-2-YL)PHENYL][(PYRIDIN-2-YL)AMINO]METHYL}QUINOLIN-8-OL #1	31.7374	48.0231
7	2-METHYL-7-{[4-(PROPAN-2-YL)PHENYL][(PYRIDIN-2-YL)AMINO]METHYL}QUINOLIN-8-OL #2	29.8149	44.6212

Supplementary Methods Synthesis Scheme and Protocol. Branched tail oxyquinolines not available commercially were synthesized according to the method described below using Betti reaction. In an oven-dried 25 mL flask, equipped with a magnetic stirrer, corresponding aldehyde (2 mmol) and amine (2 mmol) were dissolved in dry EtOH (3 mL), and the resulting mixture was stirred at ambient temperature for 10 min. Corresponding 8-hydroxyquinoline (2 mmol) and dry pyridine (80 μL, 1 mmol) were added, and the resulting mixture was stirred for 5–20 days. Products insoluble in ethanol formed a white precipitate that was filtered, washed with ethanol, and dried under high vacuum. In the case of the products soluble in ethanol, the solvent was removed under reduced pressure, and product was purified on silica-gel flash chromatography using cyclohexane/EtOAc (0%–40%) as eluent.

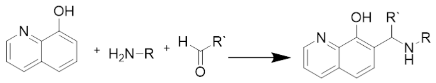

Supplement: Supplementary file 1 [file antioxidants-11-00220-s001.zip › antioxidants-1523571-supplementary.pdf]
